# Locked patellar dislocation: a case report

**DOI:** 10.1186/1752-1947-2-371

**Published:** 2008-12-04

**Authors:** Frederick Michels, Nicole Pouliart, Dirk Oosterlinck

**Affiliations:** 1 UZ Brussel, Laarbeeklaan 101, 1090 Brussels, Belgium; 2 Department of Orthopaedic Surgery, AZ Groeninge, Burgemeester Vercruysselaan 5, 8500 Kortrijk, Belgium

## Abstract

**Introduction:**

Acute patellar dislocation is a relatively common problem. The most common dislocation is laterally in the coronal plane. Sometimes spontaneous reduction occurs, but if not, closed reduction can easily be done. In this paper, we report a very uncommon type of locked dislocation which required an open reduction.

**Case presentation:**

A 16-year-old girl of Hispanic origin sustained a sudden dislocation of the patella while she was dancing. Pre-operative computed tomography revealed a patellar dislocation with rotation around the vertical axis with the patella wedged on the side of the lateral condyle. Closed reduction failed. Open reduction was needed and the torn structures were repaired. At 1-year follow-up, she had a good functional outcome and reported no recurrence of dislocation.

**Conclusion:**

This case report shows that some patellar dislocations may be irreducible with the closed technique. Computed tomography is valuable in case of doubt. If an open reduction is needed, the medial ligamentous structures should be repaired.

## Introduction

Acute patellar dislocation is a relatively common problem and most likely caused by indirect trauma (gymnastics, dancing, etc.). About 10% of acute dislocations are the result of a direct blow to the medial side. The most common dislocation is laterally in the coronal plane. Sometimes spontaneous reduction occurs, but if not, closed reduction can easily be done. In this paper, we report a very uncommon type of locked dislocation which required an open reduction.

## Case presentation

A 16-year-old girl of Hispanic origin sustained a sudden dislocation of the patella while she was dancing. There was no direct trauma involved, but just an awkward movement. Past history was unremarkable and revealed no predisposing factors (previous trauma or significant joint laxity).

**Figure 1:**
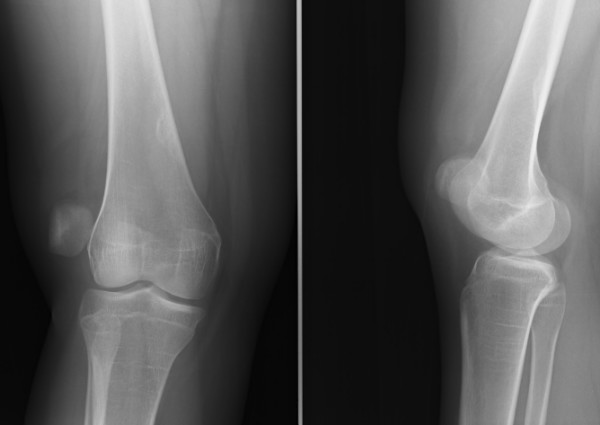
Pre-operative radiograph of the right knee showing patellar dislocation.

On physical examination, the knee was locked in extension with the patella located laterally. There was tenderness around the patellar region. A general laxity of ligaments was observed and she was moderately obese. A laterally dislocated patella was seen on plain radiographs (Figure 1).

**Figure 2:**
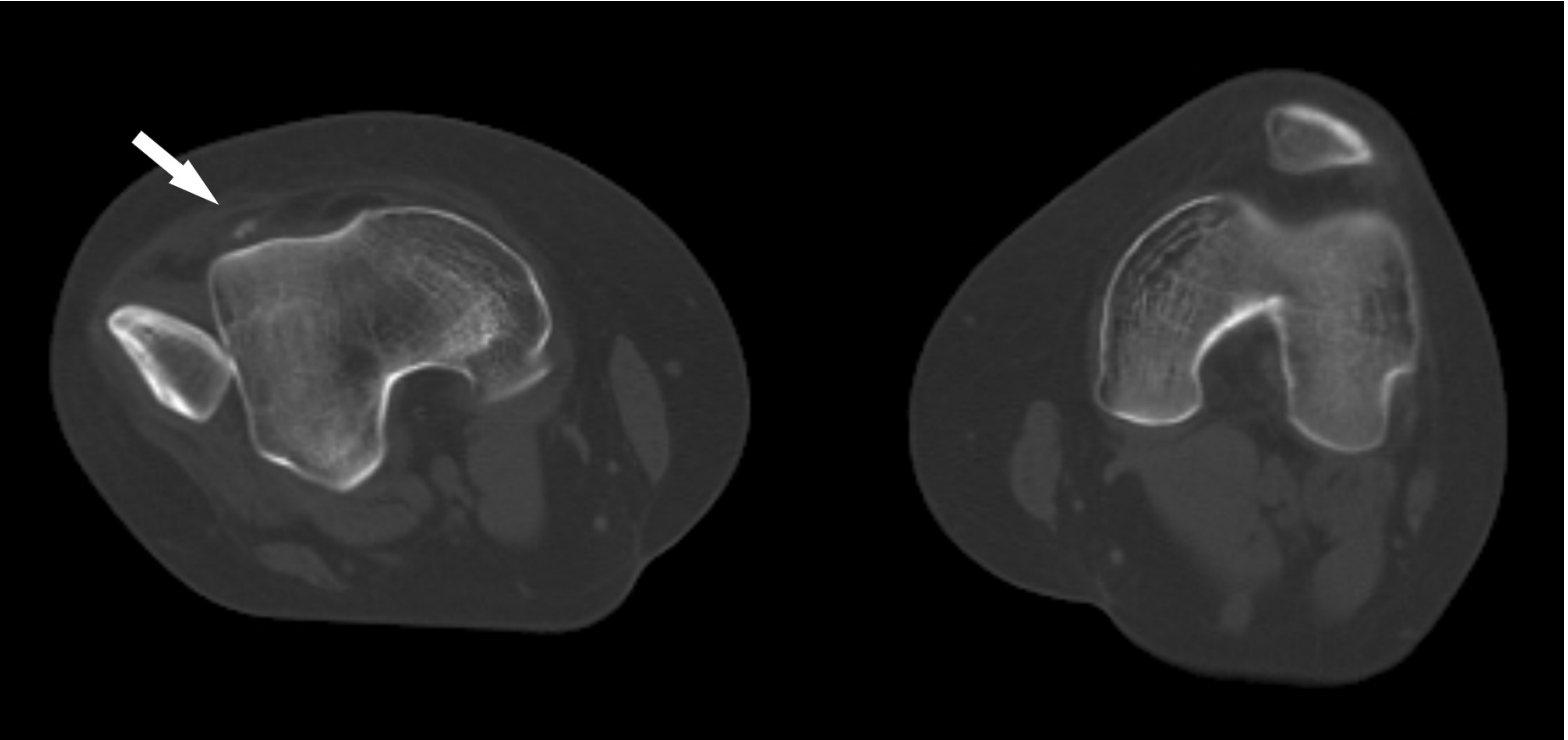
Computed tomography scan with axial image through both knees showing patellar dislocation on the right side. The arrow shows a loose body.

Closed reduction without anaesthesia was unsuccessful. A computed tomography (CT) scan revealed a laterally dislocated patella with the articular surface facing laterally and the lateral border of the patella directed anteriorly (Figure 2).

**Figure 3:**
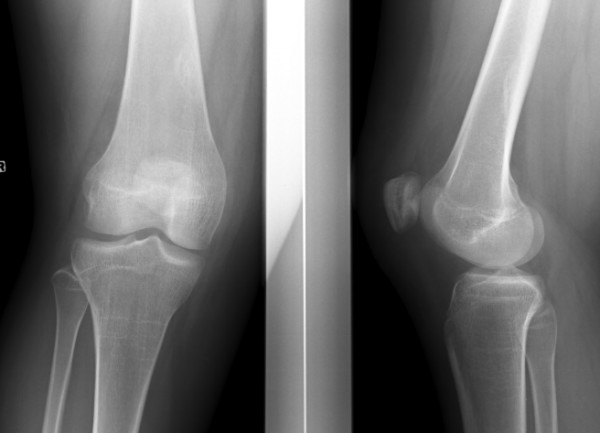
Postoperative radiograph: the normal patellar-femoral relationship has been restored.

Another attempt at closed reduction, this time under general anaesthesia in the operating room, was again unsuccessful. Surgical exploration through an anterior incision showed an important tear of the medial retinaculum and the patella appeared rotated around its vertical axis. Reduction was achieved easily by reaching under the patella and pulling it anteriorly. The patellar cartilage was intact. A loose body was resected. The medial capsule was plicated and the tight lateral retinaculum was released. Postoperative radiographs documented that the patella was in the correct position (Figure 3). The patient received a cylindrical cast postoperatively for 2 weeks, after which physiotherapy was started. Six months postoperatively, she had regained a very good function and was able to take up dancing again. At 1-year follow-up, she reported no recurrence of dislocation or signs of subluxation.

## Discussion

In this report, we describe a patellar dislocation with rotation around the vertical axis with the patella wedged on the side of the lateral condyle.

To avoid confusion, we recommend using the classification proposed by Ofluoglu *et al. *[1]. This distinguishes two main groups depending on the location of the patella in the patello-femoral joint. In intra-articular dislocations, the patella remains in its anatomical position and is only rotated around its vertical or horizontal axis. In extra-articular dislocations, the patella is displaced outside the patello-femoral joint. According to this classification, the present dislocation can be classified as an extra-articular lateral dislocation with rotation on the vertical axis [1].

Since the dislocation occurred while dancing, the trauma mechanism was probably a combination of internal rotation of the femur on the tibia combined with contraction of the quadriceps, followed by a flexion movement combined with an external rotation of the femur.

This exceedingly rare injury has only been reported twice before. Corso *et al. *[2] reported a lateral dislocation with vertical axis rotation in a 16-year-old boy by a laterally directed blow to the patella while wrestling. ElMaraghy *et al. *[3] reported a similar dislocation in a 30-year-old woman caused by a hyperflexion movement. In both cases, closed reduction failed and open reduction was required.

Two similar dislocations have been reported in association with a fracture. Hackl *et al. *[4] reported a rare case of a lateral dislocation with a bony avulsion of the medial structures after a fall from a chair. The remaining medial patellar margin was impacted into the lateral femoral condyle and the patient required an open reduction. Gidden and Bell [5] reported the case of a 15-year-old boy who was involved in a motorcycle accident causing a high-energy trauma to the knee. The patella was irreducible with vertical axis rotation and the medial border forced into the femur, causing a Salter-Harris III physeal fracture. Open reduction of the patella and internal fixation of the lateral condyle with two compression screws were necessary.

This is the first report where this lesion is confirmed by CT scan. As half of the reported cases were associated with fractures, we deem that a CT scan is valuable in case of doubt.

As this type of dislocation represents a major trauma to the knee with extensive damage of the medial ligamentous structures, open reduction offers the additional opportunity of reconstructing these ligaments. It also allows inspection of the joint and removal of possible loose cartilage bodies [3]. In this patient, the lateral retinaculum was also divided, whereas this was not done by ElMaraghy *et al. *Both techniques appear to yield good results [3].

## Conclusion

This case report shows that some patellar dislocations may be irreducible with the closed technique. A CT scan is valuable in case of doubt. If an open reduction is required, the medial ligamentous structures should be repaired.

## Consent

Written informed consent was obtained from the patient for publication of this case report and any accompanying images. A copy of the written consent is available for review by the Editor-in-Chief of this journal.

## Competing interests

The authors declare that they have no competing interests.

## Authors' contributions

FM was the attending physician who was responsible for diagnosis and treatment. FM and NP analysed and interpreted the patient data. FM drafted the manuscript. NP performed a critical revision of the manuscript. DO made a substantial contribution to conception and design. He also performed a critical revision of the manuscript. All authors read and approved the final manuscript.
